# Sharing decisions amid uncertainties: a qualitative interview study of healthcare professionals’ ethical challenges and norms regarding decision-making in gender-affirming medical care

**DOI:** 10.1186/s12910-022-00880-y

**Published:** 2022-12-27

**Authors:** Karl Gerritse, Casper Martens, Marijke A. Bremmer, Baudewijntje P. C. Kreukels, Fijgje de Boer, Bert C. Molewijk

**Affiliations:** 1 Department of Ethics, Law and Humanities (ELH), Amsterdam University Medical Center (UMC), Location VUmc, De Boelelaan 1089a, 1081 HV Amsterdam, The Netherlands; 2 Center of Expertise on Gender Dysphoria (CEGD), Amsterdam UMC, Location VUmc, De Boelelaan 1118, 1081 HZ Amsterdam, The Netherlands; 3 Department of Psychiatry, Amsterdam UMC, Location VUmc, De Boelelaan 1118, 1081 HZ Amsterdam, The Netherlands; 4 Department of Medical Psychology, Amsterdam UMC, Location VUmc, De Boelelaan 1118, 1081 HZ Amsterdam, The Netherlands

**Keywords:** Gender incongruence, Transgender, Ethical challenges, Ethics, Shared decision-making

## Abstract

**Background:**

In gender-affirming medical care (GAMC), ethical challenges in decision-making are ubiquitous. These challenges are becoming more pressing due to exponentially increasing referrals, politico-legal contestation, and divergent normative views regarding decisional roles and models. Little is known, however, about what ethical challenges related to decision-making healthcare professionals (HCPs) themselves face in their daily work in GAMC and how these relate to, for example, the subjective nature of Gender Incongruence (GI), the multidisciplinary character of GAMC and the role HCPs play in assessing GI and eligibility for interventions. Given the relevance and urgency of these questions, we conducted a qualitative study among HCPs providing GAMC to transgender adults in the Netherlands.

**Methods:**

In this qualitative research, we conducted 11 semi-structured interviews between May 2020 and February 2021 with HCPs (six mental health professionals, two HCPs in endocrinology, two in plastic surgery, and one in nursing) working in two distinct GAMC settings. We purposively sampled for professional background and years of experience in GAMC. We analyzed our interview data using thematic analysis. As some respondents were more inclined to speak about what should or ought to be done to arrive at good or right decision-making, we identified both ethical challenges and norms. Furthermore, in our analysis, we differentiated between respondents’ explicit and implicit ethical challenges and norms and ascertained the specific context in which these challenges emerged.

**Results:**

Respondents’ ethical challenges and norms centered on (1) dividing and defining decisional roles and bounds, (2) negotiating decision-making in a (multidisciplinary) team, and (3) navigating various decision-making temporalities. These themes arose in the context of uncertainties regarding (1) GAMC’s guidelines, evidence, and outcomes, as well as (2) the boundaries and assessment of GI.

**Conclusions:**

This interview study provides detailed empirical insight into both the explicit and implicit ethical challenges that HCPs experience and their ethical norms regarding decision-making. It also describes how uncertainties and (implicit) normativities concerning GAMC and GI pre-structure the moral environment in which these challenges and norms manifest. We provide normative reflections and recommendations on handling these ethical challenges in a way that is sensitive to the context in which they arise.

## Background

A marked increase of transgender^1^ and gender diverse individuals seek gender-affirming medical care (GAMC), e.g., interventions such as feminizing and masculinizing hormones and/or surgery to aid the affirmation and expression of their experienced gender [1]. Since the late 1970s, an international group of clinicians, professionals, and other stakeholders have worked diligently to develop best practices to promote the health and well-being of transgender clients. Although the clinical population has changed considerably over the years (now including, e.g., youth and non-binary individuals), these efforts have resulted in consensus regarding the standards of care and the guiding ethical principles of GAMC for adults [2, 3]. However, like in other care contexts, healthcare professionals (HCPs) providing GAMC are inevitably confronted with various ethical challenges [4–7]. We define ethical challenges as situations in which a stakeholder asks oneself, does not know, is in doubt, is uncertain, or disagrees with another stakeholder about what is right or good [8, 9]. In previous qualitative research [4], we identified six themes around which HCPs experience such challenges that we will here relate to key characteristics of GAMC.

First, although the evidence base is growing, current clinical guidelines for GAMC are primarily based on expert opinion leaving many clinical questions (e.g., regarding long-term follow-up and risks) unanswered [2, 3]. The latter gives rise to or complicates ethical challenges in determining who should be rendered eligible for GAMC (Theme 1) and establishing what constitutes good GAMC (Theme 2). Second, GAMC often comprises various interventions requiring different multidisciplinary professionals' involvement, [2] leading to ethical challenges in multidisciplinary cooperation and regarding the sequential order of treatment (Theme 3). Third, the growing diversity of trans individuals, identities, and treatment requests [10] and changing demographic characteristics [11, 12] generate, amongst others, ethical challenges concerning the role of clinical guidelines and whether these ought to be guiding or prescribing (Theme 4). Fourth, the object of care is currently classified as “Gender dysphoria” (GD), a mental disorder in the fifth Diagnostic and Statistical Manual for Mental Disorders [13], but also as ‘Gender Incongruence’ (GI), a condition related to sexual health in the 11th revision of the International Classification of Diseases [14]. This points to ongoing shifts in and divergent understandings of the clinical conceptualization of gender diversity and the object of care [15]. Consequently, assessing GI/GD can be clinically and ethically challenging [16] (Theme 5).

The abovementioned culminates in the final theme: decision-making (Theme 6). Indeed, HCPs may face ethical challenges regarding shared decision-making with clients and how to organize (multidisciplinary) decision-making in GAMC. Examples of such challenges include: How should I share the responsibility for decision-making when a client suffers from co-occurring mental health concerns, which makes me doubt their ability to consent to treatment? Or: In a triad consisting of a surgeon, mental health professional (MHP), and client, who ought to have what kind of responsibility regarding the acceptability of risks involved with surgical treatment? This paper centers on challenges in GAMC for adult transgender clients (i.e., those aged 18 years and above).

Ethical challenges regarding decision-making are further complicated by MHPs often playing a pivotal role in GAMC generally and decision-making specifically. According to the 7th version of the Standards of Care (SOC7)^2^ of the World Professional Association for Transgender Health (WPATH), MHPs are best prepared to diagnose GI and establish eligibility for GAMC as well as to guide clients throughout their gender-affirming process given their specific training and as medical treatment is intensive, often life-long and (partially) irreversible [2].

This role, however, is not without its challenges. MHPs themselves, for example, struggle with the question of to what extent they (should) have a guiding or assessing role in decision-making [4, 18]. Concurrently, debates concerning their decisional role and decision-making approaches in GAMC are polarizing. On the one hand, many HCPs and transgender activists argue that the role of MHPs in decision-making effectively renders them “gatekeepers,” curbing trans clients’ right to self-determination [19, 20]. This critique has led to the development and implementation of alternative care models that seek to minimize the involvement of the MHP in decision-making and foster a more liberal individual account of “client autonomy” through so-called “Informed Consent Models” for GAMC for transgender adults. On the other hand, legislation and other efforts aimed at or curbing the provision of GAMC to especially trans youth is on the rise, undergirded, amongst others, by claims that current decision-making practices insufficiently safeguard the principle of non-maleficence [21, 22]. The latter often highlight the risk of “detransition” (i.e., discontinuing and/or reversing GAMC) and the more general clinical challenge of accurately identifying for whom treatment is beneficial or harmful [23, 24].

Against this backdrop, the paucity of empirical work investigating HCPs’ ethical challenges in decision-making regarding GAMC for adults is surprising. Much empirical literature on ethical challenges encountered by HCPs in GAMC focuses on care for trans youth [25–28] and specific interventions such as fertility preservation [29]. Some studies do not focus solely on decision-making [4] or ethical challenges in decision-making per se [16, 18, 30, 31]. Both Dewey [18] and shuster [16], however, identified challenges pertaining to (the implementation of) collaborative decision-making in GAMC. MacKinnon et al. [30, 31] found that HCPs face challenges in using assessment protocols, establishing “transition readiness,” and preventing “regret,” especially in clients diagnosed with complex mental health issues. Taken together, the literature suggests that ethical challenges related to decision-making in GAMC are ubiquitous, relevant, and urgent but remain understudied. Notably, studies seeking to understand these ethical challenges through the experience of HCPs are absent. So are those appreciating these ethical challenges in relation to the particular context of GI and GAMC.

Therefore, we initiated a qualitative interview study into the ethical challenges experienced by HCPs regarding decision-making in GAMC for adult clients. We included a majority of MHPs (HCPs with a background in psychology or psychiatry) as their involvement in decision-making is central and contested. To account for the multidisciplinary nature of GAMC, we also included HCPs with other professional backgrounds (i.e., endocrinology, plastic surgery, nursing). The research question was: What ethical challenges related to decision-making do HCPs face in their daily work in GAMC?

This study aims to contribute to various goals, including (1) better understanding HCPs' ethical challenges related to decision-making in the specific context of GI and GAMC; (2) informing various stakeholders about these challenges; (3) identifying barriers and facilitators for recent calls from a variety of stakeholder groups to implement shared decision-making in GAMC [32, 33]; (4) reflecting on the question as to what good decision-making in GAMC should entail. Ultimately, this study seeks to improve decision-making practices and the handling of ethical challenges related to decision-making in GAMC.

## Methods

We conducted a qualitative interview study to explore the ethical challenges of HCPs regarding decision-making in GAMC. We selected a qualitative methodology because it provides room for detail and in-depth information. It allowed us to pay attention to the fine nuances of the ethical challenges experienced and expressed by HCPs [34, 35].

### Setting

Dutch GAMC is offered by three multidisciplinary University Medical Centers (UMCs) and, increasingly, nonacademic mental healthcare centers that often work in partnership with UMCs and other somatic healthcare providers. Dutch GAMC guidelines largely follow WPATH’s SOC7 [2] and are adapted to the local infrastructural, legal and professional context. For this study, we recruited HCPs at an academic and nonacademic center participating in a larger project on ethical challenges concerning (shared) decision-making in Dutch GAMC (2019–2022).

### Participant selection and recruitment

We included HCPs with a minimum of one year of working experience in GAMC. To meet the criterion of maximum variation, we purposively sampled for professional background and years of experience [34]. Recruitment took place by asking a gatekeeper, in this case, a member of the steering group of the larger project, to bring us in contact with possible respondents. This person informed and provided a list of potential respondents from the participating academic center, of which we approached ten via e-mail. Another steering group member contacted a nonacademic GAMC center's stakeholder, who proposed two participants based on our in- and exclusion criteria. The names used in this writing are pseudonyms.

### Data collection

KG conducted nine interviews, and CM and BM each conducted one semi-structured interview. In six interviews interviewer and respondent were not acquainted with each other, while in the other five, the interviewer and respondents knew each other as (in)direct colleagues. We based our interview guide on previous empirical and conceptual research [4, 5], the abovementioned literature, and our experiences and observations as clinicians and CES staff in GAMC. We purposefully abstained from providing theoretical definitions of ‘decision-making’ and ‘ethical challenges’ as we wanted to elicit respondents’ concrete experiences. Our final interview guide included open-ended questions, e.g., What ethical challenges related to decision-making do you experience in your daily work? Can you give an example of a case or situation in which it was hard to come to a decision or where the decision-making process felt wrong or uncomfortable to you? Conversely, can you sketch a case or situation in which the decision-making process felt particularly right or good? After conducting two interviews, we discussed the interview technique and guide among the research team. The interviews were audiotaped, transcribed verbatim, and anonymized.

### Data analysis

We analyzed the anonymized transcripts according to a thematic analysis approach [35]. First, KG read the transcribed data and listened to the recordings to ensure the accuracy of the transcription and foster data immersion. Second, fragments relevant to the research question were coded inductively in MaxQDA 12.0 employing open codes, which entails that we coded all potentially relevant textual fragments. We emphasized respondents’ original wording (in-vivo coding). KG and CM independently coded two transcripts resulting in an initial code list. They compared their code lists, reached a consensus, and resolved discrepancies through dialogue. Using and adding to this initial code list, KG and BM independently coded the third transcript and reached a consensus, resulting in a code system that KG, MB, and BK used to code independently and discuss the fourth transcript. KG drew from this last code system to code the rest of the dataset while adding new codes. Third, KG and CM convened to cluster codes to identify initial (sub)themes they discussed with the other authors. Fourth, further coding by KG took place to ensure no codes had been missed in the earlier stages (to a total of 239 codes and 1147 fragments). Furthermore, KG and CM re-coded the last three transcripts to allow for a ‘deductive check’ of the (sub)themes. During this process, KG and CM refined the (sub)themes which they subsequently discussed with the other authors. We reached data saturation: we did not find underexplored (sub)themes during data analysis or identify new codes during our deductive check [35]. The last stage involved selecting relevant quotes to illustrate the identified (sub)themes.

In the absence of an agreed definition, clear methodological guidelines, and consensus in social scientific and empirical ethics literature, it is challenging to identify ‘ethical challenges’ in empirical qualitative data [8, 36]. We developed the following approach. First, following Molewijk et al. [8] we defined ‘ethical challenges’ as situations in which a stakeholder (a) asks oneself whether one does the right or good thing; (b) does not know what the right thing to do is; (c) is uncertain or in doubt about what is the right or good thing to do; (d) disagrees (with another stakeholder) about what is morally right or good to do; (e) knows what is right or good to do but is not able to or allowed to do that; or (f) feels obligated or forced to do something which one believes to be morally wrong or bad. As we found that some HCPs were more inclined to speak in terms of what should or ought (not) to happen to arrive at good or right decision-making, we also included ‘ethical norms’ in our analysis. Subsequently, we differentiated between explicit and implicit ethical challenges and norms. Explicit challenges and norms are those verbalized by our respondents. We identified Implicit ethical challenges as those that HCPs (seemingly) use without intention and/or are unaware of [37]. Furthermore, we distinguished ethical challenges and norms from the ‘context’ they pertained to. For example, the context of “having co-occurring psychiatric problems” often corresponded with the explicit ethical norm “we shouldn’t rush decision-making.” This approach proved a useful heuristic device which we will elaborate on in the Findings and Discussion sections. To enhance the credibility of our findings, we engaged in recurring reflexive dialogues among the research team and conducted two member checks [35]: we presented and discussed our findings during a policy day of HCPs in Dutch GAMC, and member checked our findings with participating MHPs in the context of the co-creation of an ethics support tool (we report on the development process of this tool in a separate manuscript).

### Research team

The research team consisted of a trained ethicist, qualitative health researcher, and Ph.D. candidate who was also a junior M.D. working in GAMC at the time data collection took place (KG), a healthcare consultant, and community advocate (CM), a senior researcher, and psychiatrist working in GAMC for adults (MB), a senior researcher in medical psychology focusing on gender identity development and (outcomes of) GAMC (BK), an expert in qualitative health research (FdB) and an ethicist and senior researcher with experience in clinical ethics support (CES) in GAMC (BM). We are a group of different genders and sexualities. To foster reflexivity, we engaged in dialogues among the research team members about how our professional and personal positionalities impact our assumptions, relationships with respondents, and research decisions [38]. Furthermore, an advisory group and steering group consisting of client advocates and academic/clinical experts (some of whom identify as transgender or non-binary) offered practical and methodological input for this study.

## Results

Out of the 12 HCPs we approached, 11 agreed to participate in the study, and one did not reply. We conducted 11 interviews between May 2020 and February 2021, nine of which took place via Microsoft Teams due to the COVID-19 pandemic. Information about respondent characteristics can be found in Table 1.

**Table 1:** Characteristics of the research respondents

Respondent*	Institution	Professional background	GAMC Experience	Interview duration
Jasper	Academic	Mental health	< 5 years	69 min
Maria	Academic	Mental health	5–10 years	58 min
Ellen	Non-academic	Mental health	< 5 years	55 min
Marieke	Non-academic	Mental health	< 5 years	54 min
Stefan	Academic	Mental health	10 + years	60 min
Wil	Academic	Mental health	10 + years	80 min
Ellis	Academic	Endocrinology	< 5 years	58 min
Wietske	Academic	Plastic surgery	< 5 years	57 min
Tim	Academic	Endocrinology	5–10 years	72 min
Mike	Academic	Plastic surgery	5–10 years	69 min
Sara	Academic	Nursing	5–10 years	74 min

^*^Names are pseudonymized

We identified three main themes. Respondents’ ethical challenges and norms centered on (1) how and when not to share decision-making with clients, (2) negotiating decision-making in a (multidisciplinary) team, and (3) navigating through various decision-making temporalities. These themes arose in the context of uncertainties regarding (1) GAMC’s guidelines, evidence, and outcomes, as well as (2) the boundaries and assessment of GI/GD.

### How should we divide and define decisional roles and bounds?

HCPs expressed ethical challenges and norms regarding the following aspects of the client–clinician decision-making process: (a) determining client–clinician decisional roles, (b) MHPs’ gatekeeper role, and (c) delaying or withholding treatment. The overarching ethical challenge in this theme was: How should we weigh respect for clients’ self-determination against our duty to non-maleficence?

#### What ought to be my role in the decision-making partnership?

Generally, HCPs strive to form a team with their clients and seek to work towards a shared goal. Some HCPs engage in a meta-conversation to discuss this explicitly. For example:

At the start of someone’s trajectory, I always tell my patients, ‘We’re a team, and as a team, we’re going to figure out how your dysphoria works for you, but especially whether medical steps will contribute to your happiness.’ I feel that whenever possible, I just want to stand by a patient’s side, form a team that works towards whatever that patient wants, but also see whether that’s sensible. (Jasper, MHP)

Here, we see that the ideal of “forming a team,” “standing next to someone,” and “working towards the clients’ goal” has its boundaries. Indeed, Jasper also expressed an ethical obligation to assess whether the client’s goal is sensible and likely to contribute to their happiness. An implicit ethical question we identified here is: Who should define “happiness,” and what it entails for decision-making in GAMC?

As Maria shared, the commitment to respecting self-determination and informed consent in decision-making may come into conflict with one’s professional responsibilities:

In our multidisciplinary team meeting this morning, we discussed a case in which, well, there were some concerns. At the same time, one of our colleagues rightly said, ‘Yes, but there’s obviously informed consent.’ And, you know, someone has to have the ability to decide for themselves and have a say in, well, what they want in terms of [medical] steps. And if someone can do that [i.e., give informed consent], who are we to say that we’re not going to treat? On the other side, there’s your responsibility, of course: your responsibility as a psychologist or your medical responsibility as a doctor. That can be pretty complicated when I think a patient may want something very much, but it just doesn’t sit right with me. (Maria, MHP)

Many MHPs described how they adapt decision-making roles and responsibilities to their clients, for example, by distinguishing between “relatively good functioning” and “complex” clients or those who are and are not able or willing to communicate. Maria shared how her role in decision-making becomes more paternalistic when confronted with clients with ‘questionable capacity’ or those unwilling to seek treatment for interfering mental health concerns. Defining one’s role and responsibilities in decision-making may also create ethical uncertainty. Consider, for example:

Interviewer: What isn’t black or white?

Jasper: Well, the road to happiness. In this case, how sure should we be of our assessment that hormones will do this patient [with suspected co-occurring mental health concerns] good? Which obstacles and hoops should this patient jump through before we can do that? And sometimes, that’s very clear. So, you’ll say that someone has to be in mental health care, and well, they might live with their mother, so in that case, the mother has to be somewhat on board with it, too. Well, then, you know what you’re working towards. But a lot of the time, it’s more ambiguous. … How far should you go? (Jasper, MHP)

#### How should MHPs relate to their gatekeeper role?

We can better understand Stefan’s ethical uncertainty in the context of MHPs having dual ethical obligations in decision-making. Many MHPs, like Maria, explicitly spoke of how their responsibilities regarding guidance may come into conflict with those regarding assessment, or gatekeeping:

As a clinician, you have a strange role. On the one hand, you try to stand by your patient’s side to find out, like, ‘What do you need? And what is necessary for you to take that step towards medical treatments here?’ On the other hand, you’re indicating clients for treatment and deciding when that’s happening. (Maria, MHP)

MHPs were often cognizant of the effects their gatekeeping role can have on (the possibility of) forming a client–clinician partnership in decision-making. Here is Stefan:

I don’t think it’s good when patients feel like I’m an obstacle they must overcome. … I want to have a position in which I’m taking them along or guiding them in their trajectory in an expert role, but with the client as a second expert or something. But at the same time, these two [roles] are at odds … because in the end, you as a clinician, well—in the context of our team-based approach, of course—have to say something about whether there is or isn’t Gender Dysphoria and whether or not we should treat. (Stefan, MHP)

The above can give rise to a situation in which clients—due to pragmatic motivations or mistrust—may approach their MHP instrumentally, e.g., by refraining from divulging information MHPs consider important in decision-making. Stefan, for example, recounted a client who did not tell him about a dissociative identity disorder diagnosis out of fear of being rejected for mastectomy. As Marieke emphasized, such a breach of trust in the client–clinician relationship can have serious ethical consequences for (the quality of) decision-making: “When a client doesn’t trust me because he’s afraid that I’ve something to decide … I also can’t determine what’s going on or what he needs” (Marieke, MHP).

#### When should we delay or withhold treatment?

Finally, HCPs expressed ethical challenges and norms related to going against clients’ wishes by withholding or delaying treatment. Although HCPs generally considered this undesirable, many shared the normative view that one should always be able to withhold or delay treatment. To ethically justify this, clinicians often refer to the principle of non-maleficence:

You know, you’ve made an oath that you want to do good and that you shouldn’t harm. Sometimes that’s... When somebody requests a treatment that I believe will do more harm than good, I’ll explain that and won’t go along. (Ellis, Endocrinology)

HCPs expressed myriad reasons for delaying or withholding treatment, including doubt regarding the assessment of GI/GD, the conviction that co-occurring mental health concerns ought to be treated or monitored before and/or during GAMC, insufficient social and/or psychological resilience, and serious concerns about a client’s ability to consent to treatment. Besides the normative view that one may only go against the wishes of the client when this is sufficiently substantiated and in their best interests, HCPs hold that they should communicate their rationale for doing so. The following quote illustrates how the latter may make shared decision-making possible even when the client’s treatment request is not (yet) granted:

Yesterday, for example, I was able to explain and motivate very clearly why I didn’t refer someone to an endocrinologist. And that person understood it, was able to follow it, and got it, too. She was disappointed, but we could talk about it with each other. And she agreed with the advice [to seek mental health care] and was going to organize it. And then I think: it’s great that we could accomplish that together. (Marieke, MHP)

### How should we negotiate decision-making as a (multidisciplinary) team?

As in many healthcare contexts, decision-making in GAMC involves many stakeholders beyond the classic client–clinician dyad. HCPs with different disciplinary backgrounds have to relate to their clients, colleagues, teams, institutions, professional organizations (and their guidelines), and the broader socio-cultural-legal context. HCPs experienced ethical challenges, particularly in (a) determining their specific responsibility in multidisciplinary decision-making and (b) handling (multidisciplinary) decision-making dissensus.

#### What should be my specific responsibility in multidisciplinary decision-making?

As Mike explained, decision-making in GAMC consists of different tasks or elements that are shared among various professionals and disciplines:

It is very different if you [as an MHP] are exploring with a patient whether a particular diagnosis fits their situation or whether you [as a somatic HCP] are discussing with someone whether we’ll perform a specific surgery. … That’s shared decision-making of a completely different caliber. (Mike, Plastic Surgery).

The latter entails that decision-making in GAMC encompasses a variety of multidisciplinary processes and responsibilities. Indeed, HCPs distinguish between ‘psychological’ and ‘medical/somatic’ duties in decision-making that tie into but are also distinct from each other:

As a [somatic] medical doctor, I feel I should only prescribe something if I can support it and believe it will benefit someone. And, the way I see things is that it’s just really great that a psychologist has already determined whether treatment is the right step for a client. And that I, based on [the MHP’s] advice combined with my endocrinological point of view, get to decide whether to start treatment. (Tim, Endocrinology)

Tim holds that he should only initiate treatment when he agrees with it. In reaching this judgment, however, he appears to rely heavily on the MHP’s assessment, highlighting the interrelated nature of various (multidisciplinary) decision-making processes. An implicit ethical question we identified here is: How should these different (multidisciplinary) decision-making processes be integrated, and what professional/discipline should carry what responsibility?

#### How should we handle (multidisciplinary) decision-making dissensus?

MHPs considered it crucial for the multidisciplinary team to agree with a treatment decision they reached with their clients:

I’ll explain [to my client] that the team has to be on board. So, there can be a situation where I’ll tell a patient, ‘Yes, to me, it’s clear, but I wonder how I’m going to sell it to a team of medical doctors and psychologists who don’t know you.’ (Jasper, MHP)

This fragment illustrates Jasper’s anticipation of potentially differing views regarding decision-making between him and the multidisciplinary team, the implicit normative assumption being that there ought to be general support from the team for a treatment decision. This may play into a state where MHPs are reluctant to share certain case specifics with their team. As Will shared:

[T]o some extent, what I’ll share in a [multidisciplinary] team meeting, I’ll filter to what I think people want to hear. And that’s what I call the peculiar paradox of a gatekeeping model I take to my meetings. … That I guess I shouldn’t say too much. … I’m convinced that my omitting, or telling some details differently, will lead to another decision. Well, it’s not always clear-cut, but my strategizing is increasing, and I find that pretty annoying. (Will, MHP)

Conversely, diverging normative convictions regarding decision-making may be acknowledged, understood, and discussed:

We weren’t really on the same page about how to continue [in the case of a client with co-occurring psychiatric problems who suffered from complications following phalloplasty]. The surgeons said, ‘Well, should we even perform surgery again?’ And I understood why they found that difficult when looking at his resilience and how he dealt with his complications, especially considering that a second surgery carries the same—or perhaps an even higher—risk of complications. On the other hand, I felt that because we’ve said ‘A,’ we ought to say ‘B,’ too, because otherwise, we would’ve just left him hanging. … So, it felt tough that we didn’t try it one more time, while I really understood my colleagues' arguments. (Maria, MHP)

In grappling with whether performing surgery (again) is ethically permissible, Maria has to weigh her commitment to values such as trustworthiness against the surgeons’ reference to non-maleficence. In this specific case, this balancing act is made more difficult by four contextual factors, i.e., a high complication risk, the severity of suffering, the client’s co-occurring mental health concerns, and, as this MHP mentioned, his failure to understand the surgeons’ hesitance to perform surgery again. This quote also illustrates how HCPs and MHPs may engage in different decisional relationships with their clients that correspond with differing responsibilities, values, and norms. An implicit ethical challenge we identified here is: How should these diverse relationships and corresponding obligations, values, and norms be integrated into (the various steps of) the decision-making process?

### How should we navigate between various decision-making temporalities?

In the quotes above, various temporalities carry implicit or explicit normative valence in decision-making. Ethical challenges arose, particularly around (a) potential future concerns in current decision-making and (b) potential future consequences of treatment on values relevant to decision-making.

#### How should we grapple with (potential) future concerns in current decision-making?

GAMC encompasses a variety of potential treatment steps (e.g., masculinizing hormones, mastectomy, genital surgery) that are conceptualized as separate modalities with different psychosocial and physical eligibility criteria but also function as parts of a whole. In practice, this can lead to ethical challenges, for example, regarding the ‘stepwise’ approach to decision-making:

Well, I always try to keep the big picture [of the client] in the back of my mind. But the way we decide about treatment, so when you’re talking about decision-making, is really step-by-step. … We approve this step, and the client can’t derive any rights for future treatment from that approval. But OK, when I know that there’s a clear wish for further treatment, I’ll consider that. I find that very complicated now and then because I’ve patients who say from the get-go, ‘Just give me those hormones, then at least I have something,’ while I worry: How are we to move forward from there? I’ve three or four [clients who have started hormone treatment] in my caseload who are still not eligible [for surgical treatments] five years down the line. (Maria, MHP)

Consequently, Maria feels she has an ethical duty in decision-making to prevent clients from getting “stuck in the middle.” Hence, like Ellis, many HCPs found it “very important… that the timing of the start of treatment is right” (Ellis, Endocrinology). Yet, determining the right timing may pose challenges. Marieke, for example, shared how she experienced difficulty deciding whether a client with co-occurring mental health concerns should seek psychological care before starting GAMC and who should determine this. In handling this ethical challenge, she explicitly took into account potential future consequences of (her role in) decision-making: “What’s worse? Referring someone for hormones when you’re worried that person will become even more unstable, or postponing [hormone treatment] and having that make them unstable. That’s a really tough call sometimes” (Marieke, MHP).

#### How should we do justice to (potential) future consequences of treatment?

Wietske explains how she anticipates the way time, treatment, and lived experience may change clients’ values and preferences regarding said treatment:

When you take the example of a relatively young person who has always felt a great aversion towards their genitals, and they say: ‘Well, I don’t want to have sex; I want a shallow vagina [i.e., vulvaplasty],’ then that’s possible, but I find it complicated when it concerns someone in their twenties. … Look, you can’t just deepen a vagina during a second surgery … And if you’ve had an operation that causes your aversion to subside, you may start to think differently about sex. And then you’ve decided on a situation you might not have been able to imagine. In those cases, I don’t say ‘no,’ but I give extensive information about the pros and cons, and I want someone to think it through before we decide. Look, I’m not talking about someone convinced and says, ‘I don’t want to take risks, and I’m overweight and in my sixties, and I just can’t be bothered.’ To me, that’s a different story than someone who’s 25. So, what I tend to do in these situations when someone is 25, is I’ll give them very detailed information, and I’ll say, ‘You’ll have another consultation with a surgeon, and then you’ve thought about it, and you can decide together.’ And then I don’t decide but postpone. (Wietske, Plastic Surgery)

Wietske appears to face the following ethical question: What should be the impact of potential future consequences of treatment on (my role in) decision-making in the here and now? In grappling with this question, she refers to various temporalities carrying normative valence: possible treatment outcomes, effects, calendar age, and the prospective preclusion of other surgeries. Ultimately, she handles this ethical question by thoroughly informing clients, involving more stakeholders in decision-making, and employing yet another temporality, i.e., delay.

We identified how many ethical challenges and norms described arise in a context characterized by uncertainties regarding (a) GAMC and (b) GI/GD. Indeed, HCPs in GAMC have to navigate various uncertainties and corresponding contestations concerning the object of care and its treatment.

### Uncertainties regarding GAMC

We found that diverse HCPs related their ethical challenges and norms regarding decision-making to uncertainties and contestations related to GAMC, particularly its guidelines, evidence, and outcomes.

#### Uncertain guidelines

Many HCPs, like Mike, believe it is important to establish limits to decision-making through the use of guidelines, for example, through stringent criteria concerning BMI and smoking:

Setting boundaries as a clinician is essential. You know, a BMI of 30 is a BMI of 30 and not a BMI of 30.5. When someone smokes, they have to stop, and you shouldn’t be like, ‘It’s OK; two cigarettes won’t make much of a difference.’ No, you should treat everyone the same. Don’t set a precedent, … [but] within these boundaries, there are many options. (Mike, Plastic surgery)

This quote exemplifies how HCPs may invoke eligibility criteria to substantiate and solidify ethical norms regarding decision-making (i.e., we should treat likes alike/not set a precedent), marking both real and perceived boundaries of decision-making.

However, other HCPs question the certainty of such guidelines, highlighting how firm boundaries may give rise to ethical uncertainties. Sara, for instance, challenges the criterion that clients are only eligible for genital surgery after a year of hormone therapy:

You start to work here, and this is the guideline we use, which is, of course, European and worldwide. … [B]ut it makes me think, like, why do we take this route, and why is it a year and not nine months? Or a year and three months? Why don’t we tailor it to the client’s needs? (Sara, Nursing)

Sara’s ethical question could be formulated as: Are current guidelines curtailing client involvement in and personalization of decision-making ethically justifiable?

#### Uncertain evidence

Often, HCPs seek to refer to (biomedical) evidence to support and justify criteria, guidelines, and treatment decisions. Although the field of trans medicine is working towards increasing its biopsychosocial knowledge base, many clinical questions remain unanswered. The latter may lead to contestation in the client–clinician decision-making relationship and ethical challenges regarding decision-making:

We don’t have any literature on these specific lab results and corresponding risks. So, I tell my client, ‘We just don’t have the evidence!’ And she asks, ‘But then, what are you basing your recommendations on?’ So, I say, ‘On our guidelines; we have to stick to our guidelines. Studies have found that long-term exposure to high [hormone] levels can lead to problems.’ And she just couldn’t do anything with that because she found it too general. She stuck to her position and said, ‘No, I’m not going to lower my dose.’ And I wondered, where does my responsibility end, and where does the patient’s begin? … What I’m leaning towards is that if I’ve clearly explained the risks, and she still decides to use more, then that’s her responsibility. (Tim, Endocrinology)

Tim grapples with ethical questions such as: Who should carry the ethical responsibility for the potential risks of elevated hormone levels? An implicit question we identified here is: What, if any, should count as sufficient (biomedical) evidence to warrant or necessitate a different approach to this decision-making disagreement?

#### Uncertain outcomes

These questions point to another challenging characteristic of GAMC: the notion that the effects and outcomes of a treatment decision are—to some extent and especially on an individual level—uncertain and unpredictable. The latter can give rise to especially pressing practical and ethical challenges in the context of decision-making with non-binary clients:

[T]he most complicated are, of course, clients that say, ‘I want to look more [gender]neutral,’ because you just can’t with these hormones. You know, you can’t choose a bit of this and a bit of that. … So, you find yourself in a difficult situation [when a client says], ‘Yes, I do want a lower voice, and then I’ll just take the increase in hair growth for granted.’ Yes, I find that really tough, like, are we doing the right thing or not? (Jasper, MHP)

Uncertain treatment outcomes may not only complicate decision-making but also lead to ethical contestation and distress in the context of the client–clinician decision-making relationship:

What makes it difficult is that it’s not a black-and-white thing. You can’t say, ‘If you’re depressed and your mother is not on board, it’s a no.’ You can only say, ‘I reckon it’s important that you do this or that first.’ Well, of course, a patient will think, like, ‘What the fuck? You think? I think differently, and it’s my decision, so leave me alone.’ (Jasper, MHP)

We identified the following implicit ethical questions: In the absence of unequivocal evidence and individual predictors, should clinicians withhold or delay treatment to prevent potential adverse outcomes? How should the potential benefits of GAMC be weighed against its potential harms? How should these benefits and harms be defined? And who ought to weigh them?

### Uncertainties concerning GI/GD

Furthermore, we found uncertainties regarding GI/GD to impact ethical challenges in decision-making. Many HCPs referred to GI/GD as a complex problem or phenomenon. For example: “Interviewer: If one of your clients asks you, ‘What is Gender Dysphoria?’ what would you tell them? Jasper (MHP): Gosh. I’d say, ‘What a shitty question.’” Part of what makes this such a shitty question has to do with GI/GD being ambiguous and challenging to prove. Marieke (MHP) said: “When we’re talking about decision-making, the hard thing is that it’s all based on something we can’t measure.” Indeed, HCPs are critically aware of the absence of a validated marker: “Can we scan someone and say, ‘You’ve got Gender Dysphoria?’ Well, I don’t think so, I’m inclined to say” (Will, MHP). The notion that GI/GD is ontologically ambiguous and epistemically inaccessible has severe ramifications for the establishment of its boundaries and its assessment.

#### Uncertain boundaries

Questions related to the boundaries of GI/GD mainly surfaced when clinicians spoke of non-binary clients. Indeed, the diversification of gender identity/expression and the influx of non-binary clients in GAMC gives rise to ethical challenges in decision-making. Consider the following fragments:

When people just want a mastectomy, now they can. I’ve some clients who are non-binary and who suffer terribly from having breasts. But can you really make Gender Dysphoria out of that? For example, I have a client who says, ’75 Percent of the time I feel like a man and suffer from my breasts, but the other 25 I feel like a woman and then I don’t,’ who still wants a mastectomy. Well, I find that really complicated. (Marieke, MHP)

And:

The tricky thing is that some requests are hard to imagine … [like] patients who don’t want [their] nipples [placed back after mastectomy]. … Yes, well, maybe that’s due to my limited views and the fact that I tend to think in terms of ‘man’ and ‘woman.’ At the same time, there are no animals in nature without nipples. So, men and women have nipples. Why do you [i.e., the client] feel the need to be different? And so, we said, we think that’s just too odd. Some people just want to be different for the sake of being different. We shouldn’t abuse a medical transition for that. … Or people who want both a phallus and a vagina. … Are these my limitations, or is that just a really strange request? (Mike, Plastic surgery)

These fragments illustrate how uncertainty and contestation as to whether HCPs can understand a particular treatment request in the context of GI/GD may give rise to feelings of ethical uncertainty regarding decision-making. Indeed, clients requesting nippleless chests or both male and female genitals confront Mike and make him reflect on his implicit binary norms and values regarding gender identity and expression.

#### Uncertain assessment

The inability to measure, visualize, or otherwise ‘prove’ GI/GD poses obvious practical and ethical challenges. One of these is that many HCPs consider GI/GD to be something that could be something else. In other words, clients may be mistaken about their condition:

Sometimes there are people who really shouldn’t [have GAMC] but are convinced they are [gender dysphoric]; where it turns out that—in the end—it was a good decision not to start [GAMC]. People who really believe they’re gender dysphoric aren’t always. (Marieke, MHP)

MHPs mentioned “trauma,” “autism,” or “psychosis” as potential explanations for (something that at first glance may appear as) GI/GD. Many MHPs feel an ethical obligation to establish how these phenomena (inter)relate and differentiate between “authentic” or “real” GI/GD and other potential “causes.” Indeed, most MHPs held the ethical norm that as long as there is uncertainty as to whether some other phenomenon could explain GI/GD, a careful approach to decision-making is warranted. Such an approach is often anchored in the principle of non-maleficence and the corresponding norm that adverse outcomes such as regret should be prevented:

I find it important that people whose gender dysphoria is caused by psychiatric problems are identified quickly. It doesn’t happen often, but I’ve experienced it over the past years when it became clear that Gender Dysphoria, or alleged Gender Dysphoria, was caused by a psychotic disorder, for example. … And that’s bad because if the psychosis were to be left untreated … [GAMC] could lead to feelings of regret. (Stefan, MHP)

Yet, many HCPs, like Stefan, also question the possibility of genuinely diagnosing GD:

Well, you know what’s difficult is that we’re talking about identity, which is challenging to classify in terms of whether it’s there or not. And, well, how someone experiences their identity is highly subjective and, by definition, true because someone feels it that way. Yet, in the diagnostic phase, we try to assess whether that’s right. That’s what I find difficult. (Stefan, MHP)

As Will shared, the uncertainties concerning diagnosis may have consequences for the establishment of rapport and trust in the client–clinician decision-making relationship:

Well, I’ve spoken to many people over the years who ask me, ‘Look, what do you want to hear?’ To which I respond, ‘Well, your story,’ so to speak. And then people say, ‘No, you’re not! You say you are, but what you want to hear is that I’ve suffered from Gender Dysphoria for a long time; that I meet two out of seven DSM criteria because then I have the diagnosis; that I suffer tremendously; and that I haven’t felt like a man but a woman since years long past and I would’ve preferred to have been born a woman; and that I don’t have any problems, or at least not too many. That’s what you want to hear!’ (Will, MHP)

Indeed, this fragment illustrates how uncertainties regarding GI/GD may propel (largely implicit) normative assumptions about what GI/GD is or should be and, consequently, ethical challenges related to decision-making.

## Discussion

This qualitative interview study investigated the ethical challenges and norms regarding the decision-making of HCPs working in Dutch GAMC. These pertain to the following overarching ethical questions: (1) How should we weigh respect for clients’ self-determination against a duty to non-maleficence in decision-making? (2) How should we negotiate decision-making as a (multidisciplinary) team and (3) navigate various decision-making temporalities? We elucidated that these ethical challenges and norms arise in a context characterized by epistemic and normative uncertainties (and consequently, contestations among stakeholders) regarding (1) GAMC’s guidelines, evidence, and outcomes and (2) the boundaries and assessment of GI/GD. Given these distinct characteristics, making and sharing decisions regarding GAMC is arguably characterized by context-specific and inherent moral and normative dimensions [4, 6, 7].

Clients, policymakers, and professional bodies increasingly advocate the principles of shared decision-making as an ideal when more than one medically reasonable option is available, and the role of stakeholders’ values and preferences in decision-making is amplified [39, 40]. In light of the above, the appeal for shared decision-making in GAMC [32, 33] is not surprising. Conceptually, shared decision-making has its place between informative and paternalistic decision-making and stresses the importance of personalized care, client–clinician partnership, and shared responsibility for outcomes (Elwyn et al., 2016). Shared decision-making is often operationalized as a sequential and deliberative process consisting of (1) introducing choices and eliciting goals (‘team talk’), (2) comparing and weighing alternatives (‘option talk’), and (3) discussing decisional role preference and decision-making (‘decision-talk’) [39]. In what follows, we reflect on our findings and provide normative reflections and recommendations for (shared) decision-making in GAMC. Finally, we outline the limitations of our study and suggest corresponding avenues for future research.

### Maneuvering roles, duties, and moral distress in shared decision-making

The first theme illustrates how decisional roles and boundaries in the client–clinician dyad may be ethically ambiguous, with HCPs having to straddle respect for clients’ self-determination with their duty to non-maleficence. In line with Dewey [18] our respondents identified shared decision-making as a general best practice in GAMC but also expressed how they found it unattainable with, or not in the best interests of, specific clients. Indeed, our findings show how HCPs implicitly adopt various decision-making models. They may engage in informative and deliberative decision-making with so-called ‘(relatively) good functioning’ clients while opting for a more paternalistic approach vis-à-vis clients whom they characterize as “complex,” e.g., those with co-occurring mental health concerns or low social/psychological resilience [41]. This is consistent with the ethnographic findings of MacKinnon et al. [31], who found that HCPs use the term “complex” to indicate clinical and ethical doubt regarding clients’ authenticity, decision-making capacities and “transition readiness,” and consequently warrant delays and denials of GAMC. Moreover, Like others have shown in neonatology and end-of-life care [42], our findings illustrate how the call or desire for shared decision-making may lead to moral distress when HCPs feel it is not in line with (their assessment of) the client’s best interests. However, HCPs’ conception of clients’ best interests and their doubts and reasoning behind the decision to (not) share decision-making with clients often remain implicit and under-discussed with clients.

The above has consequences for shared decision-making in GAMC. Clark et al. [32] identified open communication as necessary for shared decision-making with trans youth. Its absence may constitute a breach of the client–clinician partnership and have serious ethical consequences for the quality of decision-making, the possibility of shared decision-making, and, thus, the realization of good care [4, 18]. The latter entails that the (motivations for/doubts about a particular) decision-making approach should be made more explicit, shared, and discussed with clients. The first step towards good (shared) decision-making in GAMC is to foster the clarification of the local decisional context and deliberation of stakeholders’ normative assumptions, perspectives, and preferences concerning (shared) decision-making. This is in keeping with those stressing dialogue and dialogical consensus as the moral basis for shared decision-making [43] and its guiding ethical principles, i.e., self-determination and relational autonomy [39].

### Sharing decision-making in a (multidisciplinary) team

The second theme highlights how decision-making in GAMC involves stakeholders beyond the archetypal client–clinician dyad and deals with more than a single treatment decision. Today’s GAMC combines psychosocial care, hormone therapy, and gender-affirming surgeries, often provided by multidisciplinary teams [2]. The latter poses complex ethical challenges concerning decision-making: How should multidisciplinary decision-making be shared among MHPs, somatic HCPs, and clients? Who should have what role and responsibility? These various multidisciplinary decision-making roles and responsibilities may conflict, while HCPs often seek (multidisciplinary) team consensus. Our findings show how the latter may also impact (the possibility of) sharing decisions in the client–clinician relationship. Indeed, the multidisciplinary team having final decisional authority may limit the dyad in attuning decisional roles and frustrate the requirement for open communication [32, 43]

The above has implications for shared decision-making in GAMC. First, HCPs should discuss multidisciplinary decisional roles, responsibilities, and processes. As GAMC is a dynamic field, it may benefit from iterative deliberation on questions such as who should introduce choices, elicit goals, and compare and weigh alternatives; in other words, who is involved in (shared) decision-making, when, and with what purpose [39]. Another critical question is to what extent shared decision-making in GAMC should allow for dissensus between HCPs. Given the inherent moral dimension of GAMC, stakeholders will inevitably dissent. Our findings (p.12/13) illustrate how acknowledging (multidisciplinary) dissensus and discussion of its underpinning value conflicts may aid in pinpointing what good care and decision-making entail. Therefore, sharing and developing best practices concerning identifying and handling (multidisciplinary) dissensus may prove more worthwhile than concealing it.

### The impact of time on (shared) decision-making

The third theme foregrounds the role of time in these various decision-making processes. We may best understand decision-making in GAMC as a stepwise process comprised of multiple interconnected decisional moments [4]. Our findings demonstrate how different temporalities (e.g., calendar age, waiting time, potential projected futures) normatively impact decision-making. Most notably, HCPs regularly took into account possible future consequences of treatment in current decision-making to minimize harm, such as regret. Again, this in keeping with MacKinnon et al. [30] who identified the prediction of future transition satisfaction and prevention of regret to be one of HCPs’ major organizing principles of decision-making in GAMC. However, our findings indicate that the question of what harm entails, how it is weighed against benefits, and by whom often remains unclear. Others have questioned to what extent such a consequentialist decision-making approach fits GAMC. McQueen [44, 45] for example, argues that as decisions in GAMC concern “personally transformative treatments” and have inherently unforeseeable effects, the possibility of post-treatment regret should have no bearing on the decision-making process. He proposes a more deontological approach, i.e., assessing whether the client has good reasons to want treatment during decision-making. While the HCPs we spoke to uniformly acknowledged the impossibility of foreseeing the consequences of decision-making, they utilized divergent tactics to manage or cope with it.

The above has implications for shared decision-making. First, shared decision-making presupposes that after the introduction of choices, comparison of alternatives, and the discussion of decisional roles, the client and HCP come to a shared decision. How should shared decision-making be adapted to a series of decisions that are also a part of an overarching decision-making process? We argue that these parts and processes should be elucidated to facilitate the iterative calibration of shared decision-making and decisional roles between the client, HCPs, and other stakeholders. A guiding question for practice could be: What should be our decisional roles in relation to this particular decision, and how do these relate to the overall decision-making process? Next, a fundamental challenge to be grappled with is the impact of potential future consequences, such as harm—particularly regret—on current decision-making. Exploring this question further in conceptual and empirical-ethics research involving diverse stakeholders could prove fruitful. How do various stakeholders define harm and/or regret? What should be the normative consequences of the possibility of harm/regret in the (shared) decision-making process?

### Uncertainties regarding GAMC and GI/GD

Hypotheses abound as to what underpins these various ethical challenges regarding decision-making in GAMC. These range from an inconsistent interpretation of clinical guidelines, insufficient formal education, and little institutional support for GAMC [18] to a lack of evidence regarding (long-term) risks of GAMC, uncertain expertise, and the fear of relinquishing medical power [16]. Our findings suggest that these ethical challenges arise in the context of uncertainties (and corresponding contestations) regarding GAMC and GI/GD.

#### Uncertainties regarding GAMC

The growing but relatively small evidence base of GAMC and the inherently unpredictable effects and outcomes pose an obvious challenge to a core feature of shared decision-making, i.e., weighing benefits and harms. The latter, in turn, exacerbates ethical challenges concerning decision-making. We found that HCPs responded to this differently. While some acknowledged it, others appeared to mobilize implicit or explicit normativity to contain it. As Cribb notes: ethical challenges, such as ethical uncertainty, are potentially very destabilizing in medicine as they are “pervasive and because [they may be] hard to resolve” [45, p.22]. He illustrates how they are often contained through (implicit) normativity, i.e., (unstated) assumptions regarding what is good and bad in routine practice. This is in line with our findings. Take, for example, the contestation regarding the importance of BMI criteria for specific surgical interventions (p. 15). The two quotes are arguably an acknowledgment of, and a means to contain ethical challenges regarding decision-making, respectively. Indeed, the ethical question the guidelines arguably help to control, but also prompt is how various considerations in decision-making about GAMC in those with a certain BMI (e.g., regarding the impact of GI/GD, the risk of complications) ought to be weighed and by whom.

#### Uncertainties regarding GI/GD

We observed a similar dynamic concerning uncertainties regarding the object of care: GI/GD. The inherently subjective nature of GI/GD and corresponding uncertainty regarding its assessment, as well as uncertainty as to whether a particular set of phenomena can be understood as GI/GD, may compound ethical challenges regarding decision-making. Here, too, (implicit) normativity may be mobilized to manage ethical challenges. An example we identified in previous observational research [4] is the use of the so-called “early-onset narrative,” a colloquial set of client characteristics that lodges the etiology of GI/GD in (early) childhood, implicates a stable trans identity and consequently offers reassurance to HCPs in decision-making. The surgeon who struggled to determine whether he could understand a particular non-traditional treatment in the context of GI/GD (p. 18) illustrates how normativity may function to manage ethical challenges regarding decision-making. In both examples, critical questions from colleagues, clients, or interviewers resulted in HCPs’ normativities becoming the subject of deliberation. The latter shows how (implicit) normativity may help to contain, but also prompt, ethical challenges regarding (shared) decision-making.

We concur with Cribb that (implicit) normativity “should not be lazily valorized, that is, seen automatically as either a bad thing (obscuring important ethical questions) or as a good thing (preventing an explosion of contention)” [46]. We described how (implicit) normativities regarding GAMC and GI/GD pre-structure the moral environment in which ethical challenges and norms regarding decision-making manifest. We are also amenable, however, to others such as Berger [47]. He argues that HCPs should acknowledge and discuss uncertainties to ensure shared decision-making applies to real-life clinical encounters. Given the diversity of the transgender population, and as individual tolerance for uncertainty in shared decision-making differs [48], its omnipresence in GAMC arguably necessitates a balancing act between mindful obfuscation and reflective illumination.

### The contribution of CES for (shared) decision-making in GAMC

Our respondents indicated a clear need for support in recognizing and handling (shared) decision-making-related ethical challenges. CES aims to support HCPs and clients in dealing with ethical issues in clinical practice, thereby improving stakeholders' quality of care, cooperation, and moral competencies. CES may be provided through different services (e.g., ethics consultation, ethics committee, moral case deliberation) with varying aims, methods, and theoretical backgrounds (Hartman et al., 2018). Increasingly, CES is offered in GAMC in the form of ethics consultations [49] and Moral Case Deliberation [7]. Furthermore, CES may be integrated and interwoven into daily practice [6], for example, through the co-creation of theme- and practice-specific ethics support tools [50, 51]. The findings presented above will provide the starting point for dialogue sessions with MHPs and clients aimed at co-creating a CES tool for (shared) decision-making in GAMC.^3^ It is interesting to note how several of our respondents started reflecting on, questioning, and reevaluating the moral dimension of their decision-making practice during the interviews. The latter illustrates how research can be a tool for CES in and of itself.

### Limitations and related recommendations for future research

This study is not without limitations. First, the semi-structured character of the interviews contributed to the depth of our findings as it allowed for the verbalization and identification of ethical challenges and norms that often remain implicit. However, although our conclusions corroborate a previously conducted focused ethnography [4], they cannot be considered direct reflections of the practice we sought to understand [34]. Indeed, our interview findings should be interpreted cautiously, being inevitably (re)constructed—in memory, dialogue, and at a specific time.

Second, the findings of this paper provide an in-depth exploration of decision-making-related ethical challenges and norms experienced by HCPs working in Dutch institutional contexts where clinical guidelines are currently based on WPATH’s SoC7 [2]. Qualitative research in other socio-economic, cultural, social, and geographical contexts on similar and different service delivery models [52] should be conducted to complement and contrast our findings. “Given that we found many ethical challenges pertaining to multidisciplinary decision-making, and that the Dutch GAMC decision-making often occurs in the context of a multidisciplinary team, future studies should investigate whether ethical challenges related to multidisciplinary collaboration also exist among HCPs working in individual practices, and if so, in which way.” Furthermore, the scope of future research should do justice to the breadth of actors implicated in decision-making in GAMC. Given the paucity of literature, studies on clients’ ethical challenges and norms should be prioritized.^4^

Third, the double role of two authors as both researchers and MHPs in GAMC may have helped sensitize responsiveness to practice and build rapport with respondents, but it could also have increased the likelihood of interviewer/researcher bias. To attenuate the latter and enhance the credibility of our findings, we engaged in recurring reflexive dialogues among the research team and conducted member checks.

Fourth, we acknowledge that the implicit ethical challenges and norms we describe are our interpretations of the data. The latter entails that they might not always be experienced or shared by our respondents or readers. By offering (methodological) transparency regarding our approach, we hope to provide space for constructive disagreement and dialogue.

Fifth, we stress that this paper offers a descriptive lens on these ethical challenges and norms without the ambition to settle them normatively. The latter is in keeping with our dialogical approach to ethics which strives for moral learning through joint critical engagement and reflection rather than an outsider’s moral judgment [53].

## Conclusion

The discussion of what constitutes good (shared) decision-making in GAMC is in full swing. To contribute to this discussion, we elucidated the ethical challenges and norms of HCPs, particularly MHPs, regarding decision-making in GAMC. Our findings illustrate how decision-making in GAMC is ethically complex and dynamic. It is best understood as an ongoing dynamic process constantly, yet often implicitly, negotiated among and distributed across various stakeholders, places and times. The latter defies the archetypal client–clinician decision-making dyad and the notion of a single decision-making moment.

The multidisciplinary and temporal structure of GAMC entails that decisional role(s), responsibilities, and values may be opaque and come into conflict. Furthermore, we expounded how the context of (shared) decision-making in GAMC is rife with uncertainties and corresponding contestations. On the one hand, clients’ and HCPs’ values and norms regarding treatment are ever-changing due to the diversification of treatment options and shifts in socio-cultural discourse concerning gender(diversity). On the other hand, the subjective and ambiguous nature of GI/GD complicates assessment and establishing its boundaries. Given these distinct characteristics, (shared) decision-making in GAMC is arguably characterized by context-specific and inherent moral and normative dimensions.

Consequently, ethical challenges and normative divergence are arguably inevitable. The implications of the latter should not be underestimated: our findings indicate that—particularly underacknowledged—ethical challenges may put a significant burden on the client–clinician and clinician-team relationship, (shared) decision-making, the organization of care processes and, in the end, the quality of care. This underscores the need for more awareness of and sensitivity toward the inherent ethical challenges, normativity, and contextual uncertainties regarding decision-making. We argue that working towards good (shared) decision-making necessitates the joint identification and handling of ethical challenges and an open, reflective, and ongoing dialogue between clients and clinicians and among (multidisciplinary) teams. CES seems to offer promising means towards these ends and may consequently allow for more explicitly deliberated and justified (shared) decision-making.

## Data Availability

The dataset generated and analysed during the current study are not publicly available due to privacy concerns, but are available from the corresponding author on reasonable request and with permission of the respondents.

## Abbreviations

CES: Clinical ethics support
GAMC: Gender-affirming medical care
GD: Gender dysphoria
GI: Gender incongruence
HCP: Healthcare professional
MHP: Mental health professional
UMC: University Medical Center
SOC7: Standards of care 7
WPATH: World Professional Association for Transgender Health

## Footnotes

1 We use ‘trans’ and ‘transgender’ interchangeably as umbrella terms referring to various forms of gender identities, roles and expressions that differ from those normatively expected of one’s sex assigned at birth. Not all trans people need or seek GAMC.

2 During this research, the SoC7 were in place. Recently, however, the SoC8 have been published [17]. Unlike the SoC7, the SoC8 no longer explicitly stipulate that MHPs are best prepared to diagnose GI and establish eligibility for GAMC. According to the Assessment of adults chapter, the role of the assessor is to (1) assess the presence of GI and identify co-existing mental health concerns, (2) offer information about GAMC, (3) support trans individuals in considering the effects and risks of GAMC and to (4) assess capacity and whether the treatment is likely to be beneficial.

3 We report on the co-creation process of this theme and practice-specific CES tool in a separate manuscript.

4 We will report on client’s ethical challenges and norms regarding decision-making in a different manuscript.
